# Violence Risk Assessment in Individuals With Substance Use Disorders

**DOI:** 10.62641/aep.v53i6.1954

**Published:** 2025-12-17

**Authors:** Visnja Banjac Baljak, Nera Zivlak-Radulovic, Sreten Vicentic

**Affiliations:** ^1^Clinic of Psychiatry, University Clinical Center of the Republic of Srpska, 78000 Banjaluka, Bosnia and Herzegovina; ^2^Faculty of Medicine, University of Banjaluka, 78000 Banjaluka, Bosnia and Herzegovina; ^3^Faculty of Legal Sciences, Paneuropean University Apeiron, 78000 Banjaluka, Bosnia and Herzegovina; ^4^Clinic of Psychiatry, Clinical Centre of Serbia, 11000 Belgrade, Serbia; ^5^Faculty of Medicine, University of Belgrade, 11000 Belgrade, Serbia

**Keywords:** violence, alcoholism, drug use disorders, HCR-20^V3^ scale

## Abstract

**Background:**

Most studies show that individuals who abuse psychoactive substances (PAS) have an increased risk of aggressive behavior and the degree of increased risk varies depending on the type of PAS. This study aimed to determine the risk and level of risk for committing violence among individuals dependent on alcohol and other PAS.

**Methods:**

A cross-sectional study was carried out, including a sample of N = 100 respondents with alcohol dependence and other PAS dependence. The Historical-Clinical-Risk Management-20, Version 3 (HCR-20^V3^) was used to assess the risk of committing violence.

**Results:**

This study found no significant difference in the overall scores of the HCR-20^V3^ scale between the examined groups regarding the risk of committing violence [the historical section - presence (*p* = 0.253) and relevance (*p* = 0.379); the clinical section - presence (*p* = 0.549) and relevance (*p* = 0.191); the risk management section - presence (*p* = 0.506) and relevance (*p* = 0.788)]. The results obtained for certain elements of the HCR-20^V3^ (violence, violent behavior, and violent ideation) showed statistical significance between the examined groups: the presence of violence risk (*p* = 0.042), violent ideation (*p* < 0.001) and violent behavior (*p* = 0.016) and relevance of violence risk (*p* = 0.009) and violent ideation (*p* < 0.001) was more pronounced in the alcohol dependent group.

**Conclusion:**

Our data confirm that respondents with alcohol dependence exhibit a higher risk of committing violence compared to respondents with other PAS dependence. Findings show that conducting risk assessment for committing violence among respondents with alcohol dependence and respondents with other PAS dependence is crucial, as both the healthcare system and outpatient services should focus on maintaining established abstinence and preventing relapse in terms of potential repeated violence-related behaviors.

## Introduction

The World Health Organization defines violence as “the intentional use of physical force or power, threatened or actual, against oneself, against another person or against a group or community, which either results in or has a high likelihood of resulting in injury, death, psychological harm, maldevelopment, or deprivation” [1]. When it comes to aggressiveness, gender differences are present, men are more likely to express aggressive behavior physically and/or directly, whereas women are more likely to express aggressive behavior indirectly [2]. A tendency towards aggressive behavior can be facilitated by various cultural and biological factors, such as early childhood events and life experiences, drug or alcohol dependence, genetic mutations that alter the function of neurotransmitters or their receptors, as well as neurodegenerative diseases, brain injuries, vascular lesions, and other conditions that impair the morphological-functional integrity of the brain [3]. The brain areas activated during aggressive behaviors have been deeply investigated, and several brain abnormalities leading to aggressive actions have been identified. The centers in the ventromedial, ventrolateral, and dorsolateral prefrontal cortex; anterior cingulate cortex and insular cortex; and structures of the limbic system (amygdala and hippocampus) show differences in structure, volume, or function in convicted criminals or aggressors compared to non-aggressive individuals [3, 4]. Aggressive behavior is strongly associated with the abuse of alcohol and other psychoactive substances (PAS) dependence [5]. Chronic alcohol abuse significantly affects the neural networks responsible for decision-making, self-control, rational thinking, and emotion processing. These changes increase the likelihood that a person will behave unpredictably and potentially dangerously. Neurobiologically, alcohol disrupts neurotransmitter signaling, primarily affecting gamma-aminobutyric acid (GABA) and serotonin receptors. It also induces behavioral sensitization, most likely by acting on the mesocorticolimbic dopaminergic pathway, which is also involved in sensitization to other addictive substances [3]. In addition, alcohol dependence increases activity in dopaminergic circuits in the nucleus accumbens, frontal cortex and amygdala. Alcohol may also act on the glutamatergic system within certain brain circuits to promote aggression in laboratory animals [6]. A review has shown that PAS dependence directly or indirectly increases dopamine levels in the nucleus accumbens and generally enhances activity in the mesocorticolimbic system [7]. This provides strong evidence that this system is directly responsible for the link between aggressive behavior and addiction [3, 7]. Many used drugs have complex mechanisms of action that accentuate autonomic drive, threat perception, and limbic-based behavioral responses [8]. The relationship between opioids and aggression is complex and involves multiple neurotransmitter systems, including serotonin, dopamine, and GABA, as well as neuropeptides like vasopressin and oxytocin. For instance, low levels of serotonin are associated with impulsivity and aggression, while the overstimulation of dopamine receptors can lead to paranoid and aggressive responses [9]. Some of the phenylethylamines, such as methylenedioxymethamphetamine and methamphetamine, also have serotonergic properties, with the potential for long-term neurotoxic effects on the serotonin pathway, resulting in impulsive behavior, due to insufficient top-down modulation. For instance, methamphetamine use has been associated with decreased serotonin transporter density in the orbitofrontal cortex and anterior cingulate cortex, a finding correlated with increased levels of aggression [8, 10]. Furthermore, some compounds found in marijuana have an effect on central endocannabinoid receptors that control many behavioral functions, including aggression. A review of cases of marijuana and violence has reported that panic attacks, confusion, hallucinations, suspiciousness, and paranoia often occur in chronic marijuana users, affecting their cognition in ways that enhance aggressive responses to perceived provocations [11]. The association between alcohol dependence and violence-related behaviors has been confirmed by numerous studies, whether related to harmful alcohol abuse or alcohol dependence [6, 12]. In terms of violent crimes, research has shown that there is a strong link between alcohol dependence and aggressive behaviors. This link was observed in large-scale epidemiological studies and causal effects were determined in laboratory experiments [13].

On the other hand, most studies consistently confirm that individuals with PAS dependence have an increased risk of aggressive behavior and violence, individual studies have shown that the degree of increased risk varies depending on the type of PAS [5]. Furthermore, the results of some studies indicate a correlation between violent acts and dependence on certain PAS [12, 14].

Given the tendency for violent behavior in individuals with alcohol and other PAS dependence, it is important to apply a risk assessment for violent behaviors. Risk assessment is a dynamic process, and it must be repeated and evaluated over an extended period of time to include factors related to the person’s psychopathology and environmental factors. A comprehensive assessment also implies the use of risk assessment instruments in order to reduce subjectivity and the unstructured nature of clinical evaluations [14]. The aim of our research was to assess the risk and risk levels for committing violence among individuals with alcohol and other PAS dependence, as well as to highlight the importance of applying risk assessment instruments in the healthcare system to prevent relapse of violence-related behaviors among the dependence population.

## Methods

### Respondents

This study was designed as a cross-sectional study. The research procedure was conducted from February to November 2024 at the Clinic for Psychiatry of the University Clinical Center of the Republic of Srpska (UCC RS). The study population consisted of 100 respondents divided into two groups — N = 50 individuals dependent on alcohol and N = 50 individuals dependent on other psychoactive substances (cannabinoids, opioids, psychostimulants, synthetic psychoactive substances, etc.), aged 18–65 years, male gender.

### Inclusion and Exclusion Criteria

Inclusion criteria were: respondents with a clinically and diagnostically confirmed diagnosis of alcohol dependence or other psychoactive substance dependence, along with heteroanamnestic data (from family members, police, or social work centers) regarding the respondents’s physical and/or verbal violent behavior. Inclusion was carried out successively, according to the inclusion criteria, from the first day of the study onwards, until the required number of respondents was obtained, based on sample size determination using program G*Power version software (version 3.1.9.7 for Windows 10, 2020, Franz Faul, Edgar Erdfelder, Axel Buchner, and Albert-Georg Lang, Heinrich-Heine-Universität Düsseldorf, Germany), for multivariate analysis of variance (MANOVA) [15]. Based on previous research examining domain-specific differences between substance use disorder populations [16] which reported varying effect sizes across domains (Historical: d = 0.45–0.65, Clinical: d = 0.25–0.45, Risk Management: d = 0.20–0.35), an overall multivariate effect size of f = 0.25 was estimated. Power analysis for MANOVA with three dependent variables (Historical, Clinical, and Risk Management presence scores), alpha set at 0.05, and power at 0.80, indicated a minimum required sample size of 120 respondents. The obtained sample of 100 respondents provides adequate power to detect large multivariate effects and moderate power to detect medium effects, with sufficient sensitivity to explore domain-specific differences through follow-up univariate analyses.

Exclusion criteria were: female respondents, respondents dependent on two or more psychoactive substances at the same time, respondents with psychiatric disorders such as psychoses from the schizophrenia spectrum, delusional psychoses, mood disorders, respondents with dementia or other severe organic brain disorders.

### Procedure

At the onset of the study, basic sociodemographic characteristics of respondents (age, educational status, employment status, marital status, financial status, and origin) and medical history (data on psychiatric treatment, personal and family medical history) were collected.

The risk of committing violence was assessed using the Historical-Clinical-Risk Management-20, Version 3 (HCR-20^V3^) clinical assessment tool (Kevin S. Douglas, Stephen D. Hart, Christopher D. Webster & Henrik Belfrage, 2013 by the Mental Health, Law and Policy Institute, Simon Fraser University, Canada) [17]. The study used the original version of the instrument, translated into Serbian.

### Measure and Scoring Interpretation

The HCR-20^V3^ is a risk assessment tool based on structured professional judgment and includes ten items related to personal history, five clinical items, and five items related to risk management. Historical items refer to previous antisocial and violence-related behaviors as well as a history of mental disorders. Clinical items assess adaptation to clinical conditions, while risk management items focus on expected adaptation to future circumstances. The tool uses a three-point scale (0–2, where 0 indicates no risk, 1 indicates a possibility/partial presence of risk, and 2 indicates an absolute presence of risk). Based on the scoring system, evaluators make general assessments of risk levels (0 – low risk, 1 – moderate risk, 2 – high risk). The HCR-20^V3^ is also used as a scoring instrument, where scores are summed to generate risk ranking [18]. Although the structural and conceptual framework of the HCR-20 remains consistent across editions, version 3 (HCR-20^V3^) introduces an additional requirement: the evaluation of the relevance of each risk factor, in addition to its presence. The HCR-20^V3^ comprises 20 risk factors across three domains: historical (10 items), clinical (5 items), and risk management (5 items). Both presence and relevance ratings are coded numerically (0, 1, or 2). Item scores are summed to yield a total score (range: 0–40) and domain-specific subscale scores (historical: 0–20; clinical: 0–10; risk management: 0–10). The presence of risk factors is rated as *No/absent*(not present or not applicable), *Possible presence* (partially or possibly present), or *Yes/definite presence* (clearly present). Relevance is rated as *Low risk* (minimal relevance to violence), *Moderate risk* (moderately relevant), or *High risk* (highly relevant). Thus, the presence ratings capture whether a factor exists, whereas the relevance ratings indicate the extent to which that factor contributes to risk (low, moderate, or high). For the subsequent multivariate regression analyses examining the predictive validity of presence and relevance ratings for violence risk, total scores from the historical subscale were used, as this domain encompasses the majority of violence-related items.

### Statistical Analysis

SPSS statistical software (Statistical Package for the Social Sciences, version 20) was used to perform the statistical analysis of data (IBM SPSS Statistics for Windows, Version 20.0. Armonk, NY: IBM Corp.). Microsoft Excel 2013 (version 15.0, Microsoft Corporation, Redmond, Washington, USA) and Microsoft Word 2013 (version 15.0, Microsoft Corporation, Redmond, Washington, USA) were used to draw the figures and tables. Descriptive statistical measures used in the study included the arithmetic mean, standard deviation, frequencies, and percentages. To measure data, the Kolmogorov-Smirnov normality test was used. The data with a normal distribution were presented as mean ± standard deviation (SD). Homogeneity of variance was assessed using Levene’s Test for Equality of Variances (Independent samples *t*-test). Assumptions have not been violated. For comparing the mean values of variables between two populations, an independent samples *t*-test was used. The relationship between categorical variables was examined using the Chi-square test presented by χ^2^ and Phi-coefficient presented by Φ^2^. The multiple regression analysis was used for identification of predictive variables. There was statistical significance where the probability distribution was *p* < 0.05.

## Results

### Respondents Sociodemographic Characteristics

Table 1 presents the sociodemographic characteristics of respondents. Mean age of all respondents was 45.90 ± 8.02 years, with the youngest patient being 29 years old and the oldest 64 years old. Among the respondents, 42% were single, while 30% were married. The majority of respondents had a secondary/higher level of education (81%), were unemployed/retired (65%), had an average/above average financial status (75%), and lived in an urban area (67%). Statistical analysis indicates that there was significant difference between two groups by the following data: age (*p* = 0.041), marital status (*p* < 0.001), education (*p* = 0.041) and place of residence (*p* < 0.001).

**Table 1.** **Sociodemographic characteristics of study population**.

		Number (%) of respondents					
	Profile	Alc. (N = 50)	Other PAS (N = 50)	Total (N = 100)	χ ^2^	*t*	*p*
Mean age ± SD		47.54 ± 8.70	44.26 ± 7.00	45.90 ± 8.02	-	2.077	0.041
Marital status							
	Married	24 (80)	6 (20)	30 (30)	16.038	-	<0.001
	Divorced	12 (42.9)	16 (57.1)	28 (28)			
	Single/Widow	14 (33.3)	28 (66.7)	42 (42)			
Education							
	Elementary	14 (73.7)	5 (26.3)	19 (19)	4.159	-	0.041
	Secondary/Higher	36 (44.4)	45 (55.6)	81 (81)			
Employment							
	Employed	22 (62.9)	13 (37.1)	35 (35)	2.813	-	0.093
	Unemployed/Retired	28 (43.1)	37 (56.9)	65 (65)			
Material status							
	Below average	12 (48)	13 (52)	25 (25)	0.000	-	1.000
	Average/above average	38 (50.7)	37 (49.3)	75 (75)			
Place of residence							
	Urban	21 (31.3)	46 (68.7)	67 (67)	26.052	-	<0.001
	Rural	29 (87.9)	4 (12.1)	33 (33)			

Notes: SD, standard deviation; χ^2^, Chi-square test;* t*, Independent-sample *T*-test; Alc., respondents with alcohol dependence; PAS, respondents with other psychoactive substances dependence.

### HCR-20^V3^ Scale – Total Score Across Historical, Clinical, and Risk Management Sections

Table 2 shows the total score across the historical (H) section, clinical (C) section, and risk management (R) section of the HCR-20^V3^ scale (presence and relevance) for both groups of respondents. The data are presented as mean values and standard deviations (mean ± SD). Obtained results showed that there was no statistical significance between respondents with alcohol dependence and respondents with other PAS dependence relating to committing violence and risk levels for committing violence.

**Table 2.** **Total score across Historical, Clinical, and Risk Management Sections of the HCR-20^𝐕𝟑^ (presence and relevance)**.

HCR-20^V3^ scale		Alc. (N = 50)	Other PAS (N = 50)	*t*	*p*
Historical (H) scale	Presence total ($\bar{x}$ ± SD)	10.18 ± 2.50	10.80 ± 2.87	–1.149	0.253
	Relevance total ($\bar{x}$ ± SD)	9.00 ± 2.43	9.46 ± 2.76	–0.883	0.379
Clinical (C) scale	Presence total ($\bar{x}$ ± SD)	6.42 ± 1.21	6.56 ± 1.11	–0.602	0.549
	Relevance total ($\bar{x}$ ± SD)	6.16 ± 1.55	5.76 ± 1.47	1.317	0.191
Management (R) scale	Presence total ($\bar{x}$ ± SD)	8.34 ± 1.23	8.16 ± 1.44	0.668	0.506
	Relevance total ($\bar{x}$ ± SD)	7.68 ± 1.49	7.60 ± 1.47	0.270	0.788

Notes: $\bar{x}$ ± SD, value and standard deviation; *t*, Independent-sample *T* test; PAS, respondents with other psychoactive substances dependence; HCR-20^V3^, Historical-Clinical-Risk Management-20, Version 3; Alc., respondents with alcohol dependence.

### Multiple Regression Analysis of the Presence of the Risk of Violence

When applying multiple regression analysis, which are detailed in Table 3, the analysis indicated that the sociodemographic variables are not strong predictors of the *Presence* of the risk of violence in this dataset. The model as a whole is not statistically significant, and none of the individual variables show a significant impact, F (7.92) = 0.981; *p* = 0.450. The R-squared value is R^2^ = 0.069, which means that only 6.9% of the variability in “the presence of risk” is explained by the sociodemographic variables in the model. The adjusted R-squared is slightly negative, which further suggests that the model has very low explanatory power.

**Table 3.** **Multiple regression analysis of the Presence of the risk of violence**.

Variable	B Coefficient	SE	*t*	*p*	95% CI for B
Constant	11.1876	2.886	3.876	<0.001	[5.455–16.920]
Group	0.2903	0.693	0.419	0.676	[–1.086–1.666]
Age	0.0162	0.038	0.430	0.668	[–0.059–0.091]
Education	0.6843	0.641	1.068	0.288	[–0.588–1.956]
Marital status	–0.4310	0.363	–1.187	0.238	[–1.152–0.290]
Employment	0.1133	0.485	0.234	0.816	[–0.849–1.076]
Material status	–0.6314	0.636	–0.993	0.323	[–1.894–0.632]
Place of residence	–0.9809	0.724	–1.355	0.179	[–2.418–0.457]

Notes: SE, Standard Error; CI, Confidence interval.

### Multiple Regression Analysis of the Relevance of the Risk of Violence

When applying multiple regression analysis, which are detailed in Table 4, the analysis indicated that the sociodemographic variables (age, education, employment, material status and place of residence) are not strong predictors of the *Relevance* of the risk of violence in this dataset. Marital status is the only statistically significant variable in this model, with *p* = 0.042. The negative coefficient suggests that a change in marital status is associated with a decrease in the perceived relevance of the risk of violence. Overall Model Significance is F (7.92) = 1.492; *p* = 0.180. The R-squared value is R^2^ = 0.102, which means that about 10.2% of the variability in “the relevance of risk” is explained by the sociodemographic variables in the model. The adjusted R-squared of 0.034 is also very low, which further suggests that the model has very low explanatory power.

**Table 4.** **Multiple regression analysis of the Relevance of the risk of violence**.

Variable	B Coefficient	SE	*t*	*p*	95% CI for B
Constant	11.1931	2.729	4.102	<0.001	[5.773–16.613]
Group	0.3092	0.655	0.472	0.638	[–0.992–1.610]
Age	0.0085	0.036	0.238	0.812	[–0.062–0.079]
Education	0.8157	0.606	1.347	0.181	[–0.387–2.018]
Marital status	–0.7077	0.343	–2.061	0.042	[–1.390– –0.026]
Employment	0.0303	0.458	0.066	0.947	[–0.880–0.940]
Material status	–0.9656	0.601	–1.606	0.112	[–2.160–0.228]
Place of residence	–0.8733	0.684	–1.276	0.205	[–2.232–0.486]

Notes: SE, Standard Error; CI, Confidence interval.

### HCR-20^V3^ Scale (Presence) - Differences Between Study Populations Across Individual Scale Elements

The results obtained through the analysis of individual elements on the HCR-20^V3^ showed that the presence of violence risk, violence-related behaviors, and violent ideations was higher in respondents with alcohol dependence compared to respondents with other PAS dependence. Additionally, the level of violence risk and violent ideations was higher in respondents with alcohol dependence than in respondents with other PAS dependence, respectively.

**Fig. 1.**
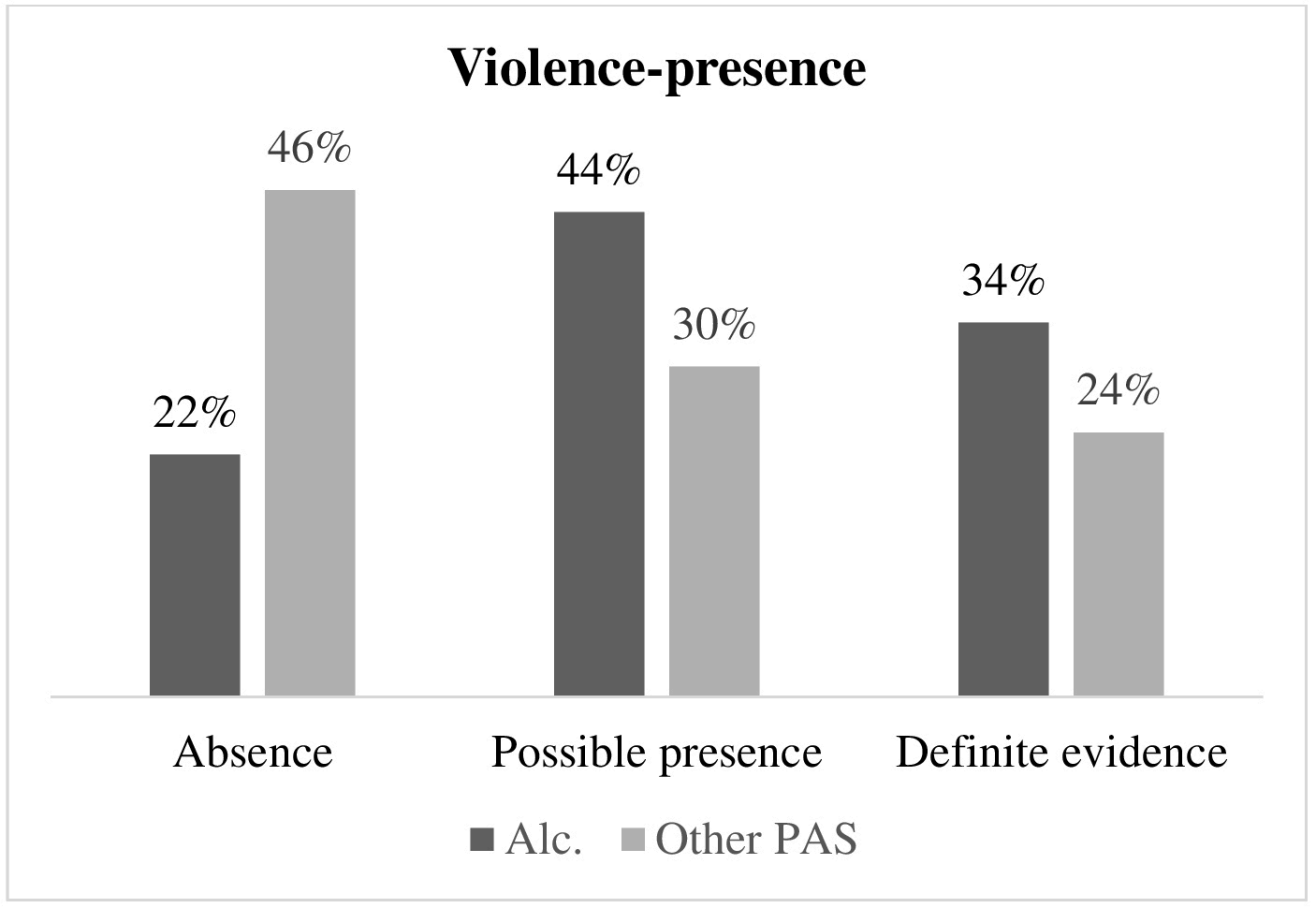
**Differences in the presence of violence in respondents with alcohol dependence and respondents with other PAS dependence**. Alc., respondents with alcohol dependence; PAS, respondents with other psychoactive substance dependence.

Fig. 1 illustrates the difference in the presence of the risk of violence between respondents with alcohol dependence and respondents with other PAS dependence. The percentage of absolute presence of violence risk in respondents with alcohol dependence (34%) was higher than in respondents with other PAS dependence (24%) (Fisher = 6.372; *p* = 0.042; Φ^2^ = 0.253).

**Fig. 2.**
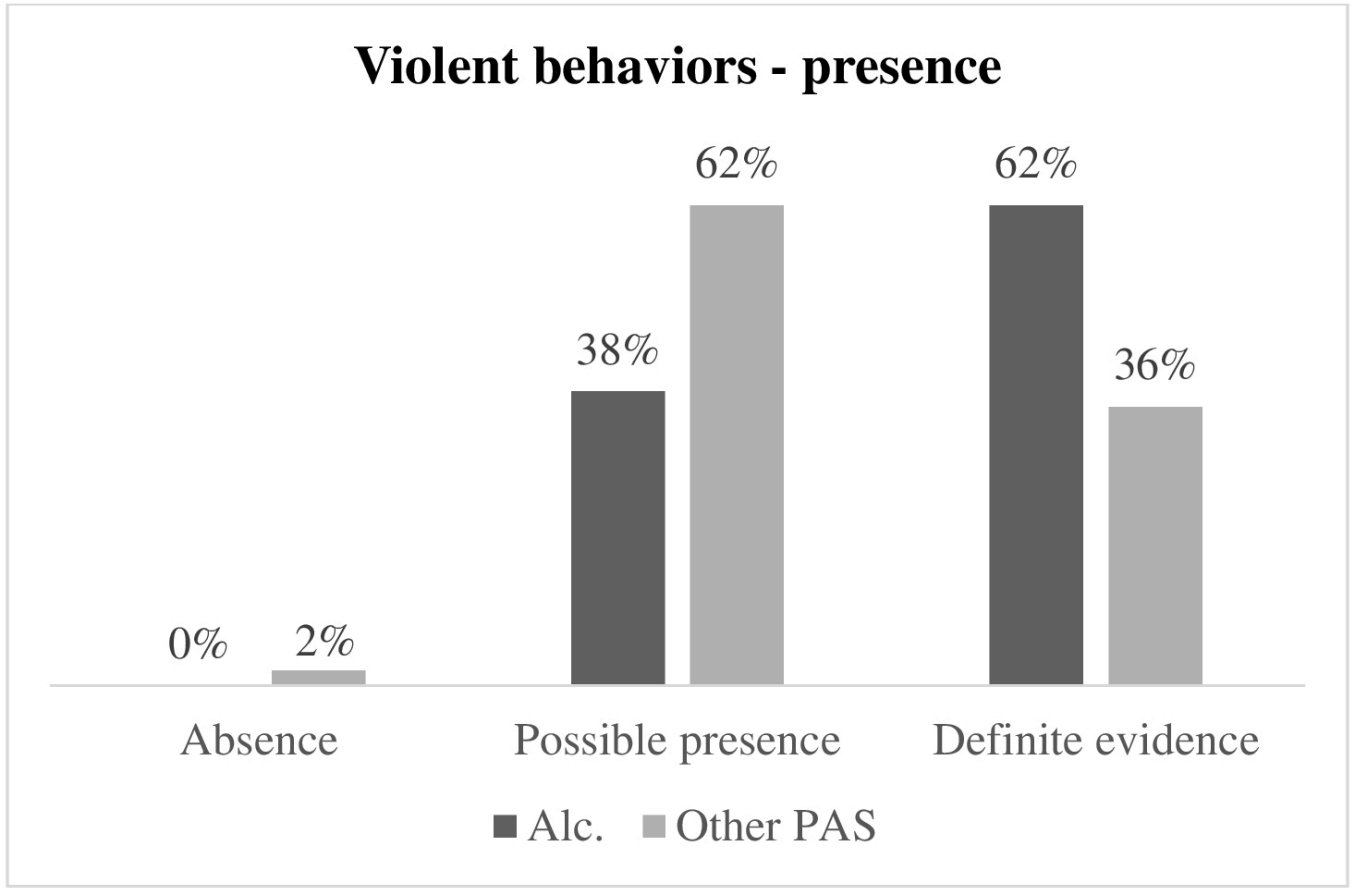
**Differences in the presence of violence-related behaviors in respondents with alcohol dependence and respondents with other PAS dependence**. Alc., respondents with alcohol dependence; PAS, respondents with other psychoactive substance dependence.

Fig. 2 illustrates the differences in presence of violence-related behaviors (enjoyment of violence or frequent acts of violence) between respondents with alcohol dependence and respondents with other PAS dependence, respectively. The percentage of absolute presence of violence-related behaviors in respondents with alcohol dependence (62%) is significantly higher than in respondents with other PAS dependence (36%) (Fisher = 7.218; *p* = 0.016; Φ^2^ = 0.271).

**Fig. 3.**
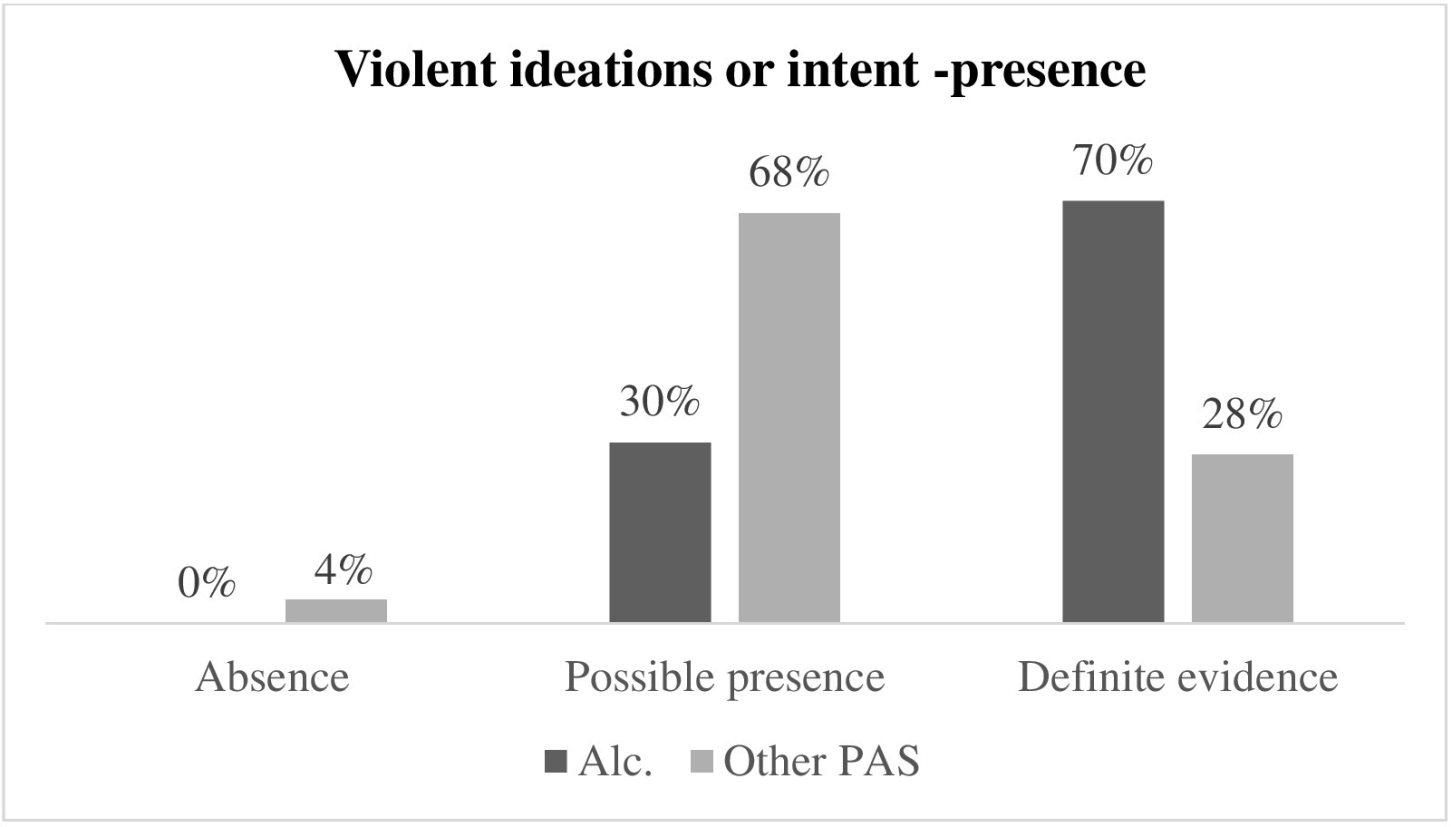
**Differences in the presence of violent ideations in respondents with alcohol dependence and respondents with other PAS dependence**. Alc., respondents with alcohol dependence; PAS, respondents with other psychoactive substance dependence.

Fig. 3 illustrates the differences in presence of violent ideations between respondents with alcohol dependence and respondents with other PAS dependence. The percentage of absolute presence of violent ideations in respondents with alcohol dependence (70%) is significantly higher than in respondents with other PAS dependence (28%) (Fisher = 18.145; *p* < 0.001; Φ^2^ = 0.429).

### HCR-20^V3^ Scale (Relevance) - Differences Between Study Populations Across Individual Scale Elements

**Fig. 4.**
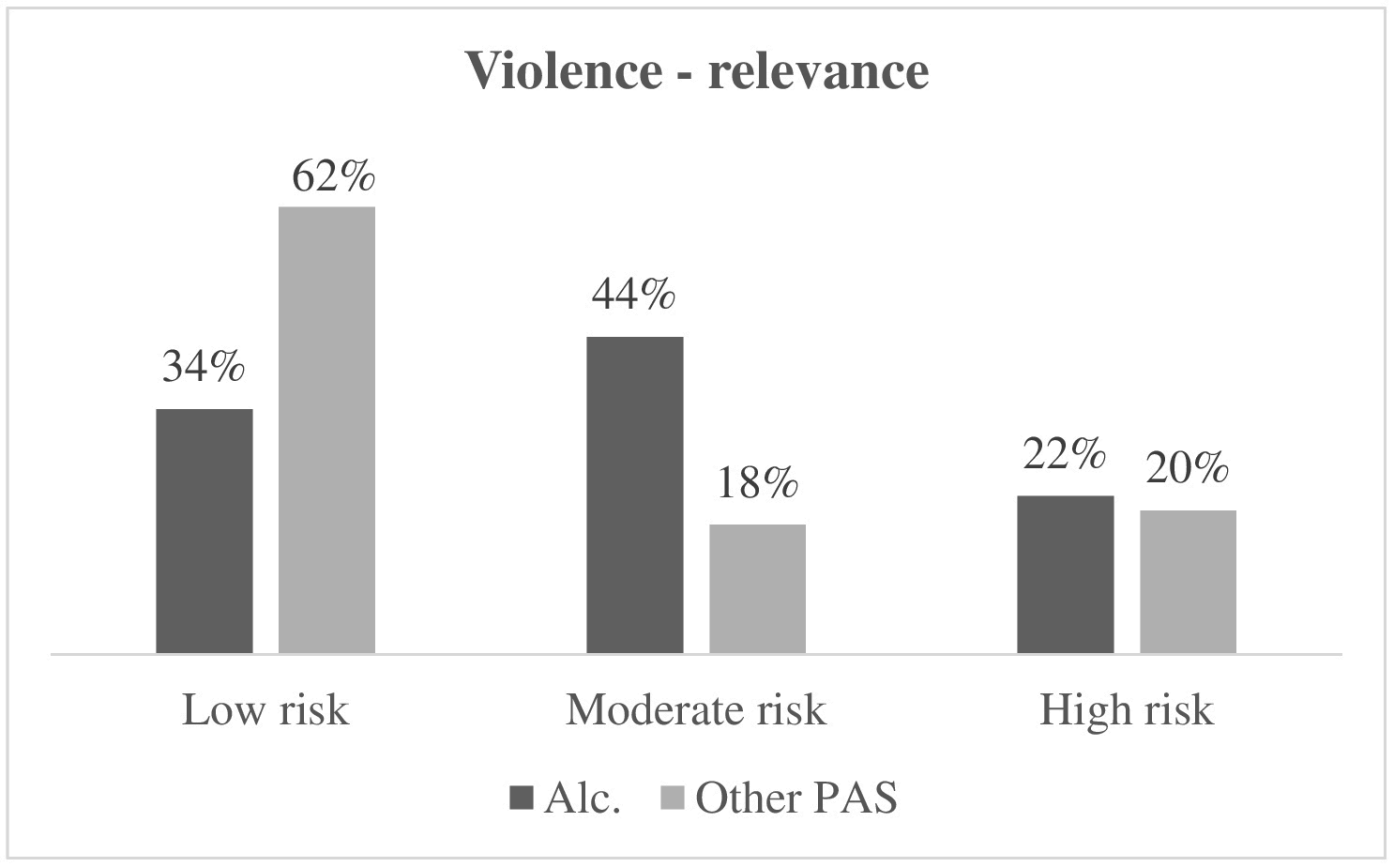
**Differences in risk levels of violent behaviors in respondents with alcohol dependence and respondents with other PAS dependence**. Alc., respondents with alcohol dependence; PAS, respondents with other psychoactive substance dependence.

Fig. 4 illustrates the difference in violence-related behaviors between respondents with alcohol dependence and respondents with other PAS dependence. The percentage of moderate risk of violence in respondents with alcohol dependence (44%) is higher than in respondents with other PAS dependence (18%) (Fisher = 9.581; *p* = 0.009; Φ^2^ = 0.310).

**Fig. 5.**
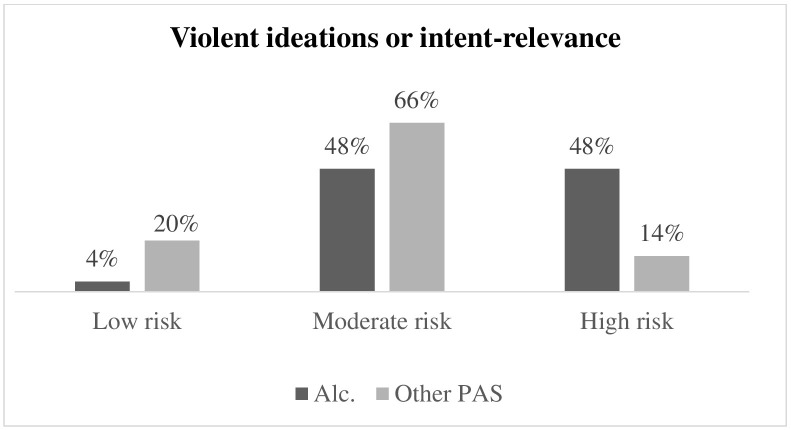
**Differences in levels of violent ideations in respondents with alcohol dependence and respondents with other PAS dependence**. Alc., respondents with alcohol dependence; PAS, respondents with other psychoactive substance dependence.

Fig. 5 illustrates the difference in the levels of violent ideation between respondents with alcohol dependence and respondents with other PAS dependence. The percentage of high risk of violent ideation in respondents with alcohol dependence (48%) is significantly higher than in respondents with other PAS dependence (14%) (Fisher = 16.250; *p* < 0.001; Φ^2^ = 0.401).

## Discussion

Considering the total score across the historical, clinical and risk management sections, findings indicate that there was no difference between respondents with alcohol dependence and respondents with other PAS dependence in regard to violence-related behaviors. However, the analysis of the results obtained across individual elements of the scale – violence presence, violence-related behaviors, and violent ideations—found that violence was more prevalent in respondents with alcohol dependence than in respondents with other PAS dependence, respectively. Additionally, the levels of violence risk and violent ideations were higher in respondents with alcohol dependence than in respondents with other PAS dependence.

As stated in research published this year individuals aged 15–49 bore the highest burden of drug use disorders, interpersonal violence, and self-harm, while those aged 50–74 had the highest burden of alcohol use disorder [19]. In our study, we identified notable differences in the sociodemographic characteristics of the respondents, particularly with respect to age, marital status, education and place of residence. The majority of respondents in our sample were middle-aged, with those diagnosed with alcohol dependence tending to be slightly older. A possible explanation lies in the fact that this trend may be partially attributed to the cultural normalization and widespread acceptance of alcohol consumption, which can contribute to delayed recognition of problematic use and, consequently, postponed treatment-seeking behavior compared to other PAS. Our data also reveal that a slightly higher proportion of individuals with other PAS dependence are single. Some other studies as well have reported a positive relationship between violence and unmarried status [20, 21]. Furthermore, in our sample the majority of individuals with alcohol dependence live in rural areas. Rates of alcohol use and alcohol-related harms vary in complex ways according to geographic area and rurality. Although those in rural communities are more likely to abstain from alcohol, among those who do use alcohol, alcohol-related harms are generally more prevalent among rural communities [22]. According to the available information, alcohol and other PAS abuse, particularly long-term abuse, significantly affects areas of the brain responsible for decision-making, rational thinking, self-control, and emotions. The changes that alcohol and other PAS induce in these regions increase the likelihood of unpredictable and potentially dangerous behaviors [2]. Aggression is a complex behavior involving interactions between the gene, physiology, environment, and personality [2, 23]. Neuroimaging studies suggested that alcohol dependence patients may share alterations in common brain regions with aggressive individuals, however, there is little known about the specific neurological mechanisms underlying aggressive behavior and whether these cause alcohol dependence patients to exhibit aggressive behavior [24]. Furthermore, dysregulation of serotonin is associated with alcohol dependence. Chronic alcohol intake increases the metabolites of serotonin in the raphe nuclei area, however reduces 5-hydroxytryptamine (serotonin) receptor 2A protein levels in the mice cortex, indicating reduced serotonergic activity [2, 25]. However, the inconsistent findings of serotonin markers in brain imaging studies of alcoholics suggest that comorbidity of alcohol dependence with other psychiatric disorders may complicate the serotonin hypothesis in real life. In addition, even individual differences in personality traits determine the types of emotion affected by the depletion of serotonin [2, 23]. In several brain imaging studies dysregulation of dopaminergic neurotransmission has been demonstrated in alcohol dependence [26, 27]. Studies investigating the interaction between genetic polymorphism of dopamine system (dopamine receptors; *DRD2*, *DRD4*, transporter; *DAT1*), and environmental factors (financial stressor and adolescent social experiences) on intimate partner violence revealed a strong influence of negative environmental changes on increased odds of violence perpetration regardless of the alleles [2, 28]. Furthermore, it has been reported that a high level of childhood adversity increases one’s likelihood to substance use through reduced functioning of the anterior cingulate cortex in inhibitory control, indicating a higher impulsive response [2, 29]. Recent models of addiction and impulsivity have focused on glutamatergic and GABAergic mechanisms in anterior cingulate cortex, given their role in impulsivity, craving and drug seeking. The elevated glutamate levels relating to an imbalance between synaptic and nonsynaptic levels are associated with dysregulation between the Prefrontal cortex and nucleus accumbens, and it was found in substance dependence. Also, glutamate levels in the dorsal anterior cingulate cortex have also been associated with delay discounting in substance use disorders (SUDs) [30, 31]. Epigenetic factors also contribute to impulsivity and addiction. In a longitudinal cohort, Wang *et al*. [32] found that impulsivity mediated the relationship between family disorganization and subsequent alcohol use, specifically amongst individuals at low genetic risk based on polygenic risk scores for impulsivity.

When it comes to violence risk assessment using the HCR-20^V3^, the available literature mainly discusses risk assessment in the forensic patient population [33, 34, 35]. There are also studies on individuals with mental disorders that were not part of the forensic population [36, 37]. However, no studies have exclusively focused on the use of the scale and violence risk assessment in the non-forensic population of alcohol and other PAS dependence. Our study confirmed that violence-related behaviors and violent ideations were more prevalent in respondents with alcohol dependence. In general, there is strong evidence linking alcohol use disorders and violence-related behaviors, despite overlaps with the abuse of other substances. One 30-year cohort longitudinal study involving N = 1265 respondents, who exhibited five or more symptoms of alcohol dependence in the previous year, found that these individuals had risk of involvement in violence that ranged from 4.10 to 11.85 times higher than those with no symptoms of alcohol dependence [38]. Most individuals with mental disorders or personality disorders who abuse alcohol are at a higher risk of committing violent crimes compared to individuals with mental disorders with no alcohol dependence. Alcohol abuse has been particularly associated with antisocial personality disorder since adolescence and has also been identified as a predictive factor for violent behavior in adulthood and criminality [2, 3]. Temperament and personality traits represent crucial factors that contribute to the development and persistence of addiction-related behaviors. Among these traits, disinhibition and lack of self-control, which are intended to be the ability to regulate one’s behavior, emotions, and cognition, represent key elements [39, 40]. In addition, aggression and violent behaviors are becoming increasingly present with the abuse of other PAS. Stimulants (such as cocaine and amphetamines) often lead to unwarranted aggression. Withdrawal crises can act as triggers for violence (conflicts within the addicted population, aggression toward family members, healthcare staff, and the police) [41]. Increased impulsivity levels have been found amongst cannabis, alcohol, cocaine and opiate-dependent individuals. Moreover, a review has shown that discounting levels vary by a type of SUDs, with particularly cannabis, opiates and cocaine being associated with most impulsivity [31]. The results of our study have identified higher levels of violence risk in respondents with alcohol dependence. Additionally, in terms of absolute risk, Swedish population data show that 8% of people with alcohol dependence and 18% of people with other PAS dependence committed violent crimes during a mean follow-up of up to 10 years [42]. Absolute rates of violent crime over 5–10 years are typically below 5% in people with mental illness (excluding personality disorders, schizophrenia, and substance misuse), which increases to 6–10% in personality disorders and schizophrenia spectrum disorders, and to more than 10% in substance misuse [43]. Several studies have shown that the risk of aggression is higher among younger patients, males, individuals with lower education levels, and addicts—especially those who simultaneously abuse multiple PAS [44, 45]. In a Swedish cohort study involving a sample of N = 49,433 male adolescents who were recruited into the military and followed for 37 years, 6% of the total cohort had committed crimes already at the time prior to conscription (18–20 years of age). Of these 5.6% had committed non-violent and 0.4% violent crimes. Also, the results of this study show that almost 60% of the total criminal group had been hospitalized for an alcohol and/or drug diagnosis [46]. One limitation of this study is that it assessed risk at a single point in time. However, risk assessment is a dynamic process, so findings and final conclusions should not be based on a single violent episode. Instead, risk assessment should be repeated and further steps should be then determined. Additionally, this study did not examine the psychometric characteristics of the HCR-20^V3^ in Serbian population. Alcohol-dependent women were not included in the study, although violence-related behaviors may also be present among them, because during the observation period, the sample consisted solely of male respondents. A relatively small sample size was not sufficient to address the potential differences among individual with PAS dependence in tendencies toward violence at different life stages. However, further research is needed.

## Conclusion

Findings obtained through the HCR-20^V3^ scale showed no difference between respondents with alcohol dependence and respondents with other PAS dependence, regarding the risk and risk levels of committing violence. However, the analysis of findings across individual elements of the HCR-20^V3^ (presence of violence risk, violence-related behaviors, and violent ideations) showed that both the risk and risk levels for committing violence are more pronounced in respondents with alcohol dependence than in respondents with other PAS dependence. Conducting risk assessment for committing violence among respondents with alcohol dependence and respondents with other PAS dependence is crucial, as both the healthcare system and outpatient services should focus on maintaining established abstinence and preventing relapse in terms of potential repeated violence-related behaviors.

## Availability of Data and Materials

The datasets generated and/or analyzed during the current study are not publicly available but are available from the corresponding author upon reasonable request.
